# *Platycladus orientalis* Leaf Extract Promotes Hair Growth via Non-Receptor Tyrosine Kinase ACK1 Activation

**DOI:** 10.3390/cimb46100665

**Published:** 2024-10-05

**Authors:** Jaeyoon Kim, Jang Ho Joo, Juhyun Kim, Heena Rim, Jae young Shin, Yun-Ho Choi, Kyoungin Min, So Young Lee, Seung-Hyun Jun, Nae-Gyu Kang

**Affiliations:** Household & Health Care (LG H&H) R&D Center, 70, Magokjoongang 10-ro, Gangseo-gu, Seoul 07795, Republic of Korea; kjy5281@lghnh.com (J.K.); janghojoo@lghnh.com (J.H.J.); juhyunkim@lghnh.com (J.K.); hina751@lghnh.com (H.R.); sjy2811@lghnh.com (J.y.S.); youknow@lghnh.com (Y.-H.C.); kimin@lghnh.com (K.M.); soyounglee@lghnh.com (S.Y.L.); junsh@lghnh.com (S.-H.J.)

**Keywords:** *Platycladus orientalis* leaf extract, quercitrin, human DPCs, hair growth, ACK1

## Abstract

*Platycladus orientalis* is a traditional oriental herbal medicinal plant that is widely used as a component of complex prescriptions for alopecia treatment in Eastern Asia. The effect of PO on hair growth and its underlying mechanism, however, have not been demonstrated or clarified. In this study, we investigated the hair-growth-promoting effect of PO in cultured human dermal papilla cells (hDPCs). *Platycladus orientalis* leaf extract (POLE) was found to stimulate the proliferation of hDPCs. POLE with higher quercitrin concentration, especially, showed a high level of cellular viability. In the context of cellular senescence, POLE decreased the expression of p16 (CDKN2A) and p21(CDKN1A), which resulted in enhanced proliferation. In addition, growth factor receptors, FGFR1 and VEGFR2/3, and non-receptor tyrosine kinases, ACK1 and HCK, were significantly activated. In addition, LEF1, a transcription factor of Wnt/β-catenin signaling, was enhanced, but DKK1, an inhibitor of Wnt/β-catenin signaling, was downregulated by POLE treatment in cultured hDPCs. As a consequence, the expression of growth factors such as bFGF, KGF, and VEGF were also increased by POLE. We further investigated the hair-growth-promoting effect of topically administered POLE over a 12-week period. Our data suggest that POLE could support terminal hair growth by stimulating proliferation of DPCs and that enhanced production of growth factors, especially KGF, occurred as a result of tyrosine kinase ACK1 activation.

## 1. Introduction

Alopecia (hair loss) is a naturally progressive disorder [1]. The disease progression of alopecia is frequently observed with senescence. Diverse factors, including disease, psychiatric disorders, mechanical stress, nutritional deficiency, hormone imbalances, and aging, cause alopecia [2,3,4]. Androgenic alopecia (AGA) is the most common type observed in males [5]. In AGA, terminal hairs are changed into thinner and shorter vellus hairs. Hair follicle miniaturization is one of the critical processes in alopecia pathogenesis; however, the underlying molecular mechanisms of AGA remain unclear. The androgen hormone, dihydrotestosterone (DHT), is the agent most suspected of causing AGA in genetic research [6].

Although alopecia is not a life-threatening disease, it has psycho-social impacts on patients, substantially impairing their quality of life [7]. Diverse strategies have been suggested for the treatment of alopecia, but the therapeutic results are limited. Two drugs, minoxidil and finasteride, have been approved and widely used for alopecia treatment by the US Food and Drug Administration (FDA) [8]. Baricitinib, a reversible inhibitor of Janus kinases 1/2, was recently approved for the treatment of alopecia areata [9]. Minoxidil, which was first developed as an anti-hypertensive drug, is efficacious in treating alopecia. Minoxidil is a potassium channel opener; however, the mechanism by which it treats alopecia remains unclear [10]. Side effects of minoxidil, such as pruritus, dermatitis, and irritation, have been reported [11]. Due to the limited efficacy of established drugs [12] and their unpredictable side effects, new therapeutic alternatives are needed for hair loss prevention and hair growth promotion.

The hair follicle undergoes three stages of development: anagen (proliferation), catagen (involution), and telogen (resting) [13]. Dermal papilla cells (DPCs), differentiated from mesenchyme stem cells and located in the core of the hair follicle, are one of the major hair cycle regulators because they control external stimuli and signals delivered through cytokines and junctions [14,15]. The miniaturization of hair follicles is represented by a depletion of the anagen stage and a reduction in hair on the androgen alopecia scalp [16]. Mesenchymal stem cell-derived signaling and growth factors from DPCs influence hair recovery and regeneration through cellular proliferation, thereby prolonging the anagen phase (KGF), stimulating hair follicle development (β-catenin), and suppressing apoptotic cues [17]. Enhancing the production of growth factors, bFGF [18], KGF [19], and VEGF [20], in DPCs could be an effective strategy for activating the hair cycle and growth.

The kinome comprises more than 500 distinct protein kinases, and it forms a unified kinase network that governs cellular phosphorylation signals [21]. In multicellular eukaryotic organisms, there are typically more than 50 different protein tyrosine (Tyr) kinases, which facilitate the phosphorylation of numerous Tyr residues across the proteome [22]. The Tyr kinome is responsible for coordinating numerous biological processes such as cell movement, cell viability, cell growth, nutrient absorption, immune response, and various stages of embryonic development [23]. The Tyr kinome has been reported to have the potential to contribute to alopecia treatment and hair growth promotion [24]. Growth factor signaling cascades, such as KGF and VEGF, are other pathways that are induced by Tyr kinome [25]. Tyr kinase ACK1, one of the targets of prostate cancer and immune regulation, has been reported to activate MAP kinase pathway and stimulate androgen receptors, which is the main target in androgenic alopecia [26]. In addition, inhibitors of Tyr kinase JAK1/2, the well-known target of alopecia areata, were approved by the FDA for alopecia treatment [27].

*Platycladus orientalis* leaf extract (POLE), “*Cheuk-Baek-Yeop*” in Korean, is a perennial herbal plant for traditional medicine that is widely distributed throughout Southeast Asia. It has been used in traditional medicine for cardiovascular disease. POLE has also been studied for its pharmacological effects: it exhibits anti-oxidative [28], anti-inflammatory [29] and anti-bacterial [30] activities. POLE is reported as an important source of a variety of natural polysaccharides and flavonoids [28]. Quercitrin, or quercetin − 3-rhamnoside, is one of the four main bioactive flavonoids in POLE. In previous research, we investigated the hair-growth-promoting effect of quercitrin [31]. Furthermore, the hair-growth-promoting effects of POLE have also been reported [32]. However, investigation into the underlying mechanisms and active ingredients of POLE with regard to the hair growth effect were limited.

We also characterized the phenolic compounds in the POLE extract. The POLE extract contains a diverse variety of chemicals. We then evaluated quercitrin, which possesses hair-growth-promoting effects, focusing on extraction lots as the major target constituent. We further investigated the physiological effects of POLE. POLE enhanced the cellular viability of cultured human DPCs (hDPCs). Furthermore, we identified the potential for hair-promoting effects through the regulation of gene expression related to the cellular senescence (CDKN1A and CDKN2A). In addition, receptor and non-receptor Tyr kinases, especially ACK1 and HCK, were also examined with POLE treatment in cultured hDPCs. As downstream targets of Tyr kinase activation, LEF−1 and DKK−1, response genes of Wnt/β-catenin signaling pathway, and growth factors (bFGF, KGF, and VEGF) were estimated. In addition, topical administration of POLE was also investigated for 12 weeks, and terminal hair density was evaluated. In this research, we found that POLE showed a hair-growth-promoting effect, which is mainly provided by quercitrin, and possesses a therapeutic potential for preventing and treating hair loss. 

## 2. Materials and Methods

### 2.1. Plant Materials and Preparation of the POLE 

The dried leaf parts of *Platycladus orientalis* were purchased from Bioeden (Nantong, China) in May 2023. The dried aerial parts of *P. orientalis* were extracted with distilled water (10 g of dried part in 90 g of DW) for 2 days at room temperature, filtered through Whatman No. 4 filter paper. The flavonoid content (quercitrin, rutin, and kaempferol) in the extract was measured using HPLC [33], and the results are shown in Supplementary Information S1 and S2.

### 2.2. Dermal Papilla Cells Culture 

**Figure 1:**
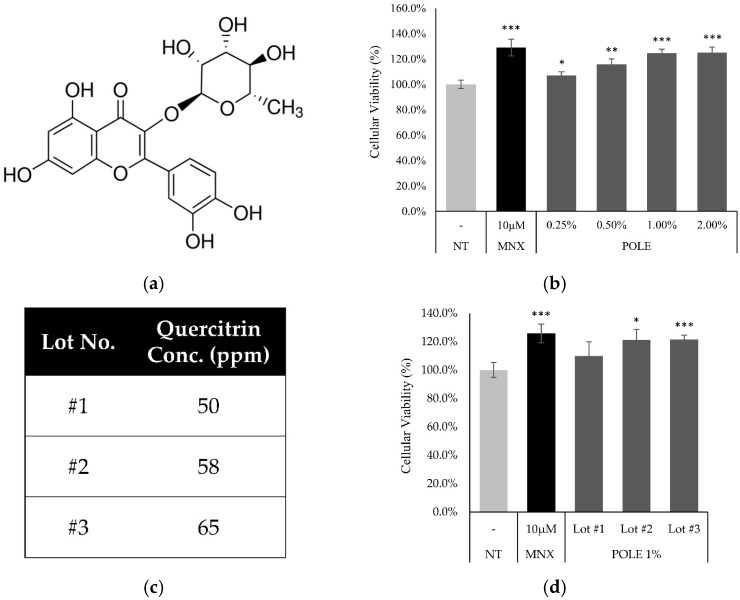
POLE enhanced cell viability in cultured hDPCs. (**a**) chemical structure of Quercitrin (Quercitrin-3-Rhamnoside (**b**) Cell viability was assessed using CCK-8 assay kit after POLE treatment (0.25, 0.5, 1, and 2%) for 24 h. (**c**) Quercitrin of POLE was examined following the lots number. (**d**) Comparison of cellular viability between POLE extracts following quercitrin concentration. N.T, non-treated control; MNX, minoxidil. Significantly different compared with N.T (* *p* < 0.05, ** *p* < 0.01, *** *p* < 0.001).

Human DPCs were purchased from Promocell (Heidelberg, Germany). DPCs were cultured in a basal medium supplemented with 4% fetal calf serum, 0.4% bovine pituitary extract, 1 ng/mL basic fibroblast growth factor, and 5μg/mL insulin (Supplement Mix, Promocell). Cells were maintained in a humidified incubator at 37 °C, with 5% CO_2_. Before quercitrin (Sigma-Aldrich, St. Louis, MO, USA; Figure 1a) treatment, serum limitation was implemented by replacing the medium with fresh DMEM (Gibco, Waltham, MA, USA) supplemented with 1% FBS (Gibco, Waltham, MA, USA) and 1 ng/mL bFGF (Merck, Darmstadt, Germany), and cultured for 24 h to minimize the effects of serum and growth supplements. Akt inhibitor API−2 and Erk inhibitor U0126 were purchased from Tocris Bioscience (Bristol, UK). 

### 2.3. Cell Viability Assay

The effect of POLE on the viability of DPCs was examined using a CCK−8 assay (Dojindo, Rockville, MD, USA) following the manufacturer’s protocols. To examine the cellular energy metabolism, NAD(P)H generation was also measured using the CCK−8 assay. The absorbance at 450 nm was read using a micro-plate reader (BioTek, Santa Clara, VT, USA).

### 2.4. Quantitative Real-Time PCR

POLE was treated at concentrations of 0.25, 0.5, and 1% for 24 h, with non-treated cells serving as a control. Total RNA was extracted using an Rneasy RNA extraction kit (Qiagen Inc., Hilden, Germany). cDNA synthesis was performed using a cDNA synthesis kit (Phillkorea, Seoul, Korea) with ThermoCycler (R&D systems, Minneapolis, MN, USA), according to the manufacturer’s protocol. cDNA samples obtained from the control and treated cells were subjected to real-time (RT) PCR analysis.

The TaqMan probes for RT-PCR used in this study were as follows: GAPDH assay id 4352934E; KGF (FGF7) assay id Hs00940253_m1; IGF−1 assay id Hs01547656_m1; LEF1 assay id Hs01547250_m1; DKK−1 assay id Hs00183740_m1; CDKN1A assay id Hs00355782_m1; CDKN2A assay id Hs00923894_m1.

TaqMan One-Step RT-PCR Master Mix Reagent (Life Technologies, Carlsbad, CA, USA) was used. The PCR reactions were performed on the ABI 7500 Real-Time PCR system according to the manufacturer’s instruction. The resulting data were analyzed using ABI software.

### 2.5. Protein Dot Blot Analysis for Growth Factor and Signal Transduction

The human growth factor antibody array kit (Abcam, Cambridge, UK), human apoptosis antibody array kit (Abcam, Cambridge, UK) and human receptor tyrosine kinase (RTK) phosphorylation antibody array kit (Abcam, Cambridge, UK), along with an anti-phosphor-tyrosine antibody, were used to elucidate the changes in growth factor profiles and signal transduction pathways in DPCs. A total of 41 growth factors, 71 tyrosine kinases, and 40 apoptosis/proliferation elements were analyzed. Cells were treated with 0.25, 0.5, and 1% of POLE for an appropriate time and then collected for growth factor analysis. Cells treated with vehicle medium were used as the non-treated control. The conventional immunoblot process was performed according to the manufacturer’s protocol. The resulting blots were analyzed under identical conditions using iBright FL1000 (Invitrogen, Waltham, MA, USA).

### 2.6. Participants and Evaluation of Hair Density

This study adhered to the tenets of the Declaration of Helsinki. The clinical studies were reviewed and approved by the Review Board of LG Household and Healthcare Ltd. (approval numbers LGHH-20221013-AB-04-01). Written informed consent was obtained from all participants before any study-related procedures. In total, 60 volunteers participated in this study. All volunteers were randomized into two groups: one group (n = 30, age: 44.5 ± 8.9) used a 1% POLE solution, while the other group (n =30, age: 45.7 ± 9.5) received a non-treated control using a computer-generated random number system to maintain allocation concealment. The investigators and participants were blinded to the group allocation. The participants were supplied monthly with identical bottles containing similar textures, colors, and smells to maintain blinding. The agents were applied via daily topical administration (1 mL/day) to the frontal and vertex regions of the scalp. The total investigation period was 12 weeks and terminal hair density was evaluated. Hair density was measured using the phototrichogram technique with ×60 magnification [10,34]. All measurements were performed by the same investigator, who was blinded.

The study followed STROBE recommendations for reporting randomized clinical trials.

### 2.7. Statistical Analysis

Statistical analyses were performed using Prism 10.0.2 (GraphPad Software, Boston, MA, USA). Data are presented as the average ± standard deviation. We assessed the data for normal distribution and similar variance between groups. Statistical significance (* *p* < 0.05, ** *p* < 0.01, and *** *p* < 0.001) was evaluated using a two-tailed unpaired Student’s *t*-test for comparisons between two groups and a one-way analysis of variance (ANOVA) with relevant post hoc tests for multiple comparisons. All in vitro experimental data are presented as the average ± standard deviation of at least three independent experiments.

## 3. Results

### 3.1. Analysis of Components of POLE

*Platycladus orientalis* leaves contain multiple types of medicinally active ingredients, including flavonoids, volatile oils, tannins, polysaccharides, and inorganic salts. The components of flavonoids in POLE were evaluated by HPLC (Table 1). The phenolic compounds (quercitrin, kaempferol, and rutin) were examined, respectively. Among these flavonoid components, the contents of quercitrin showed relatively high levels.

### 3.2. POLE Increased Cell Viability in Cultured Human DPCs

The treatment of POLE increased the viability of DPCs in a concentration-dependent manner. NAD(P)H generation, presented as cell viability, was increased by 7.1%, 15.7%, and 24.6% in the presence of 0.5, 1, and 2% of POLE, respectively (Figure 1b). In addition, following the lot number, the concentration of quercitrin was different. The increase in cellular viability from the POLE treatment was correlated with the quercitrin concentration (Figure 1d). The increments were comparable to those observed with 100 nM of minoxidil, an approved topical hair-growth-stimulating medicine and positive control.

### 3.3. POLE Enhanced Cell Proliferation in Cultured hDPCs

**Figure 2:**
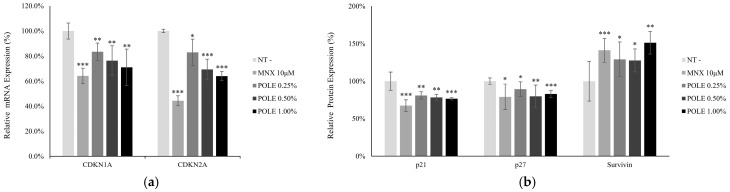
POLE modulated the mRNA and protein expression of cell proliferation and senescence in hDPCs. (**a**) The mRNA expression levels of cell cycle arresters (CDKN1A and CDKN2A) were evaluated by RT-PCR. (**b**) The protein levels of p21, p27 and Survivin were significantly changed by POLE treatment. Minoxidil was used as a positive control. MNX—minoxidil; N.T—non-treated control; significantly different compared with N.T (* *p* < 0.05, ** *p* < 0.01, *** *p* < 0.001).

Since POLE plays critical roles in cellular viability and cell survival, the effects of POLE on cell proliferation and cell cycle related markers were investigated. As shown in Figure 2, both mRNA and protein expression levels of p21 (CDKN1A) and p16 (CDKN2A) significantly decreased in a concentration-dependent manner. p21 and p16 are well-known markers of cell cycle arrest and cellular senescence [35,36]. In addition, Survivin, an anti-apoptotic protein [37], was also significantly increased by POLE treatment (Figure 2b).

### 3.4. POLE Increased Tyr Kinase Signaling Pathway

**Figure 3:**
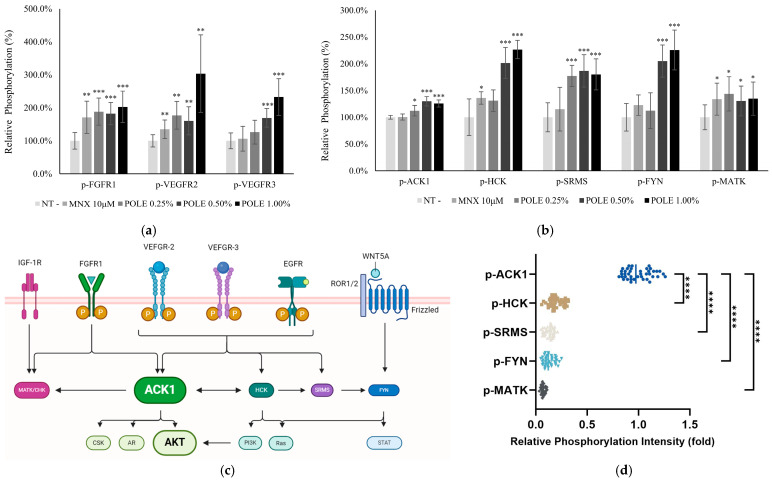
Activation of receptor Tyr kinase and Tyr kinase by POLE treatment. The DPCs were treated with or without POLE (0.25, 0.5 and 1%) for 24 h, and then collected. (**a**) The phosphorylation of three growth factor receptors (FGFR1, VEGFR2, and VEFR3) were increased. (**b**) In addition, activation of five Tyr kinases (ACK1, HCK, SRMS, FYN and MATK) were quantitated. Significantly different compared with N.T (* *p* < 0.05, ** *p* < 0.01, *** *p* < 0.001). N.T, non-treated control. (**c**) Schematic of signaling cascade for interaction between Tyr kinases and receptor Tyr kinases. (**d**) Using anti-phosphor-Tyr antibody, the relative phosphorylation of five Tyr kinases was evaluated. Significantly different compared with p-ACK-1 (**** *p* < 0.0001).

To clarify the molecular action mechanism of POLE, the phosphorylation of growth factor receptors and Tyr kinases was investigated. The phosphorylation of membrane growth factor receptors (FGFR1, VEGFR2/3) significantly increased (Figure 3a). In addition, downstream of receptor activation, Src family kinases, HCK, SRMS, and FYN, were significantly activated by POLE. ACK1, a highly activated cytosolic effector of transmembrane receptors (Figure 3d), was also significantly activated in cultured hDPCs (Figure 3b).

### 3.5. POLE Activated Wnt Target Genes in hDPCs 

**Figure 4:**
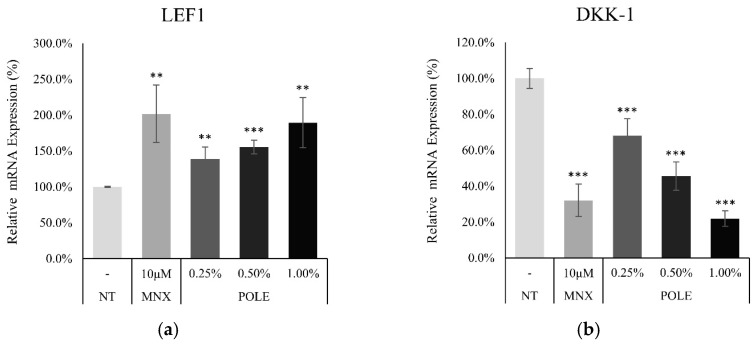
POLE regulated Wnt/β-catenin Pathway Target Gene in cultured hDPCs. The target genes of β-catenin were evaluated by RT-PCR after treatment with adenosine for 24 h. The mRNA expression level of (**a**) LEF1 was increased and (**b**) DKK-1 was decreased by POLE treatment. The data represent the means of five independent samples. NT, non-treated control; Significantly different compared with N.T (** *p* < 0.01, *** *p* < 0.001).

To elucidate the effect of POLE on downstream signaling cascade of AKT activation in hDPCs, the mRNA expression of Lef1 and DKK1 were examined after treatment with POLE for 24 h. Following the increase in Tyr kinases induced by POLE (Figure 3), the Lef1, one of the Wnt target genes [38] activated by ACK1 [39], was enhanced. In contrast, POLE also significantly decreased the expression level of DKK-1 (Figure 4), one of the major Wnt/β-catenin pathway inhibitors and telogen markers for hDPCs [40].

### 3.6. Expression Level of Growth Factors Were Increased by POLE

**Figure 5:**
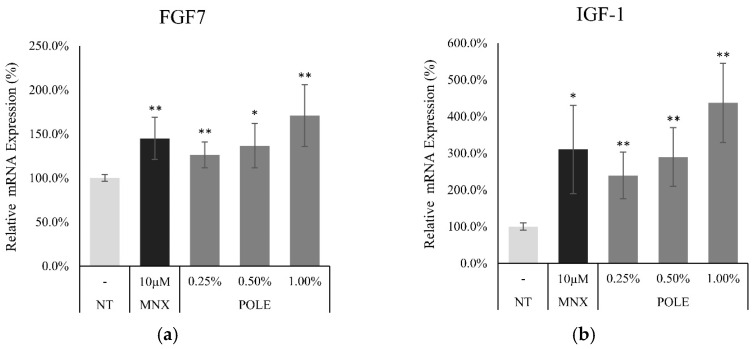
Effect of POLE on mRNA expression levels of growth factor genes in cultured hDPCs. The cells were harvested after POLE treatment (0.25, 0.5, and 1%) for 24h. The mRNA expression levels of (**a**) FGF7 and (**b**) IGF-1 gene in cultured hDPCs were measured by real-time PCR. N.T, non-treated control; MNX, minoxidil. Significantly different compared with N.T (* *p* < 0.05, ** *p* < 0.01).

The DPCs secrete a myriad of growth factors to regulate hair follicle growth. To specify the factors affecting metabolic stimulation and cell viability, the mRNA expression of growth factors and growth factor receptors was evaluated. The expression levels of KGF and IGF-1 were significantly increased by POLE treatment. mRNA expression of KGF and IGF-1 were significantly increased by 71.0% and 337.1% at 1% of POLE treatment, respectively (Figure 5).

**Figure 6:**
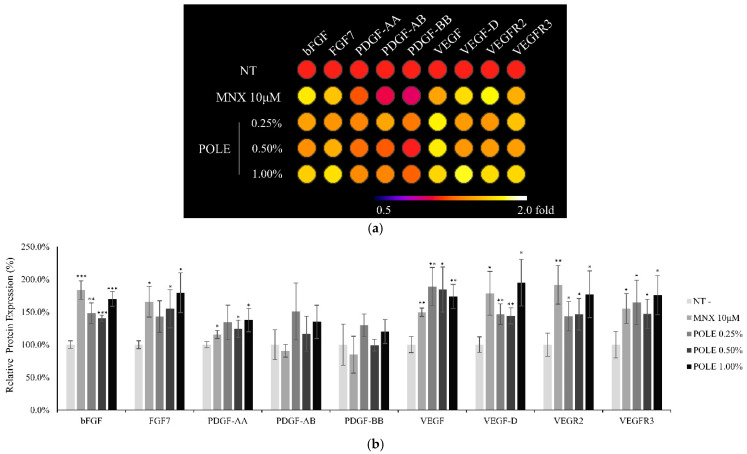
Increase of growth factor levels by POLE treatment in cultured hDPCs. The DPCs were treated with POLE (0.25, 0.5 and 1%) for 24 h, and then collected. Cells cultured with vehicle medium were used as non-treated control. The 9 types of growth factors and receptors were (**a**) displayed and (**b**) quantitated. N.T, non-treated control. Positive, biotin-conjugated IgG. Significantly different compared with N.T (* *p* < 0.05, ** *p* < 0.01, *** *p* < 0.001).

The protein expression of growth factors and their receptors to protein levels was investigated with a cultured medium. The levels of bFGF, (KGF FGF7), PDGF-AA and VEGF, which are reported to enhance the cell viability of DPCs [41], were significantly increased by POLE (Figure 6). The responsive genes by POLE were also reported as responding to growth factors of Wnt/β-catenin signaling pathway [42,43], and were found to help maintain the hair anagen stage [44,45]. Our data demonstrate that POLE enhances growth factor production through the activation of the Wnt/β-catenin pathway.

### 3.7. Hair Growth Promotion Effect by POLE Administration

**Figure 7:**
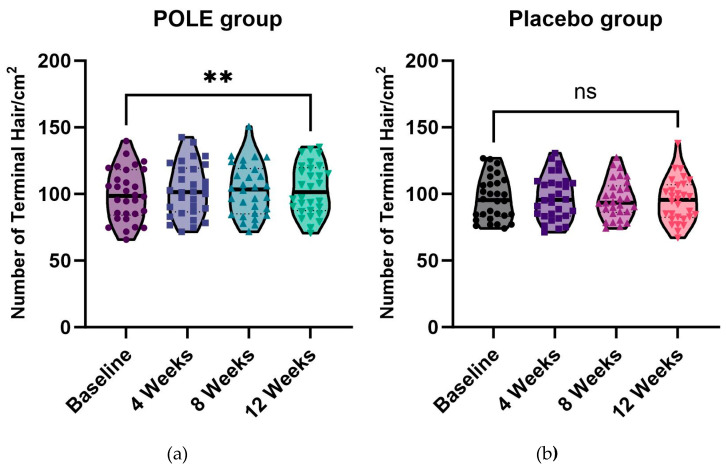
Terminal hair density changes by POLE treatment. The change in terminal hair density with the administration of (**a**) POLE and (**b**) Placebo were evaluated. Significantly different compared with the baseline (** *p* < 0.01, ns: not significant).

To evaluate the effect of POLE on promotion of hair growth, their topical administration was investigated. No significant differences in baseline characteristics, including age (*p* = 0.8288) and terminal hair density (Placebo group: 96.52 ± 16.21 number/cm^2^, POLE group: 99.21 ± 19.28 number/cm^2^, *p* = 0.5793) were observed between the two groups. At 12 weeks of administration, terminal hair density increased in POLE groups (+ 4.01 ± 6.69 number/cm^2^) (Figure 7, Table 2). However, in the control group, the terminal hair density was not significantly changed. Therefore, we propose that the effects of POLE may have contributed to scalp health and hair growth.

## 4. Discussion

The hair follicle is one of the most energy-consuming organs in humans [46]. Therefore, energy-genesis processes are highly activated in human hair follicle cells. The energy production in follicles occurs through glycolysis in the cytosol and the electron transport chain in the mitochondria. In DPCs, mitochondrial energy production strongly contributes to hair follicles [47] and affects hair growth both in vitro and in vivo [48,49]. In this study, the treatment of POLE increased cellular viability in cultured hDPCs. (Figure 1) In addition, p16 and p21, which decreased with POLE treatment (Figure 2), were reported as significant cell cycle arresters for cell proliferation. During alopecia, miniaturization of the hair follicle was observed [50,51]. The reduction of the dermal papilla volume is closely related to hair follicle miniaturization and contributes to hair thinning in the progression of alopecia [52]. Therefore, hair follicle miniaturization could be prevented by POLE treatment supporting the cellular proliferation of DPCs.

As well-known in vivo and in vitro senescence markers [53], P16 and P21, the CDK inhibitors, were also examined by POLE treatment in cultured human DPCs. P16 and P21 have essential roles in cellular senescence. Environmental stress-associated cell senescence increases the p16 expression level [54]. In addition, for cell cycle regulation among cellular senescence elements, p16ink4A (CDKN2A) and p21 (CDKN1A) inhibit associated CDKs, consequently blocking the expression of proliferation-associated genes [55,56,57].

Furthermore, the growth rates of the DPCs were different between balding and non-balding patients. The balding patients’ DPCs showed much slower growth rates than those of non-balding patients [58]. The balding DPCs are more sensitive to environmental stress and have tended to proceed to premature senescence with a higher level of p16 [59,60]. POLE decreases the expression of p16 (CDKN2A) and p21 (CDKN1A) (Figure 2). As mentioned above, our data demonstrate the possibility that POLE could support the growth of DPCs through the prevention of cellular senescence.

In addition, POLE also activated growth factors in DPCs. The growth factors activated by POLE treatment, such as bFGF, KGF, PDGF-AA, and VEGF (Figure 5), were also significantly activated by quercitrin [31]. The activated growth factors were reported to activate the hair cycle and growth [17] and to be secreted by DPCs. In particular, KGF could stimulate DPC signal cascades that participate in hair germ proliferation and a new hair cycle induction [19]. In a previous study, quercitrin was found to stimulate the production of these growth factors in cultured hDPCs [31]. It could be predicted that the growth factors contributing to prolonging the anagen stage and supporting hair growth could be supplied to the hair bulb at higher levels by quercitrin treatment. Therefore, we could suggest that POLE shows a similar mode of action to quercitrin, which could be a key active ingredient in POLE. Our data indicate that POLE could also have potential to maintain the hair follicle in the anagen state via enhancement of growth factor secretion.

POLE significantly increased the expression level of Lef1 (Figure 4), one of the major β-catenin-responsive genes that encodes a transcription factor and stimulates the anagen hair follicle stage [61]. DKK1 was reported as the major Wnt/β-catenin pathway inhibitor and telogen marker for hDPCs [40]. The production of DKK1 was also downregulated by POLE treatment. The expression of bFGF, FGF7 and VEGF, which have been previously reported to be stimulated by Wnt [42,43] and promote hair growth while elongating the anagen stage [44,45], was enhanced by POLE treatment.

In addition, in Figure 3, FGFR1 and VEGFR2/3, which is a target receptor of growth factors, such as bFGF, FGF7 and VEGF, were activated by POLE. As a consequence, Tyr kinases, such as ACK1, HCK, and SRMS, are significantly activated. The Tyr kinases activated by POLE were also significantly increased by quercitrin treatment, which is one of the candidate active molecules in POLE [31]. Notably, ACK1 is one of the well-known shuttles between the cytosol and the nucleus that transduces growth factor signals from the receptors to the intracellular effectors, such as AKT and AR [26]. In addition, activation of Src family kinases, such as HCK, SRMS and PYN, were reported to activate Wnt/β-catenin signaling [62]. Therefore, our data demonstrate that activation of Tyr kinase signaling with growth factor production could be a plausible molecular mechanism for the hair-growth-promoting activity of POLE.

Plant extracts are composed of diverse chemicals. The composition of chemicals depends on the harvest conditions of the original plants. Furthermore, the effects of the plant extract are harmonized by the chemicals. In that sense, it could be possible that the most effective concentration of plant extract, such as *Hottuynia cordata* and *Polygonum multiflorum,* varies for each phenotypical parameter [63,64]. The effects of plant extracts result from orchestrated actions, even if they are composed of the overall activities of thousands of chemicals with varying molecular signatures. Previous research has shown that quercitrin possesses hair-growth-promoting effects [31] and could be one of the candidate active components in POLE.

In this research, we found that POLE enhanced the cell viability of the human DPCs. We also found that the enhancement was accompanied by cell cycle arresters, p16 and p21, downregulation, and Wnt/β-catenin signaling activation (Lef1 enhancement and DKK1 downregulation). In addition, the proliferative effect of POLE is attributed to enhanced growth factors production, such as bFGF, KGF, and VEGF via Tyr receptor kinases such as ACK1 and HCK. Furthermore, our results suggest that topical administration of POLE exerted promotes hair growth in vivo. In conclusion, our data demonstrate that POLE stimulates hair growth by enhancing cellular viability and increasing the secretion of growth factors. We suggest that POLE could have therapeutic potential for preventing hair loss and/or promoting hair growth.

## Figures and Tables

**Table 1:** Analysis of flavonoids in POLE.

Flavonoid Name	Cas. No.	Amount
Quercitrin	522-12-3	65.2 ppm
Kaempferol	520-18-3	2.2 ppm
Rutin	153-18-4	38.0 ppm

**Table 2:** The change of terminal hair density between control and POLE group.

Group	Difference of Terminal Hair Density (Number/cm^2^)		
	Baseline	8 Weeks	12 Weeks
Placebo	-	−0.51 (±6.64)	−1.52 (±7.25)
POLE	-	+3.36 (±3.81)	+4.01 (±6.69) **

** Significantly different compared with baseline (*p* < 0.01).

## Data Availability

The raw data supporting the conclusions of this article will be made available by the corresponding author upon request.

## Supplementary Materials

The following supporting information can be downloaded at https://www.mdpi.com/article/10.3390/cimb46100665/s1, Supplementary Materials: HPLC analysis of POLE; POLE_Array Antibody Raw Data.
