# Supplementary material for: Platycladus orientalis Leaf Extract Promotes Hair Growth via Non-Receptor Tyrosine Kinase ACK1 Activation
Source: Curr Issues Mol Biol. 2024 Oct 5;46(10):11207–19. doi: 10.3390/cimb46100665 (PMC11505925; doi:10.3390/cimb46100665)
Supplement: Supplementary file 1 [file cimb-46-00665-s001.zip › cimb-3204028-Supplementary Materials.pdf]

Supplementary Materials

Supplementary Information S1. HPLC analysis of POLE

<HPLC test method>

- Agilent 1260 Infinity II system
- Column: Agilent poroshell C18 (50 × 4.6 mm, 2.7 μm)
- Column temperature: 35 ℃
- Injection volume: 3 μl
- Detector: HPLC-DAD (254 nm)

Time(min)	Flow rate(ml/min)	ACN (%)	0.2% Acetic acid (%)
0	0.6	5	95
20	0.6	20	80
40	0.6	20.5	79.5
50	0.6	35	65
90	0.6	90	10
91	0.6	5	95

Re-equilibration duration was 2 min between individual runs.

Number	Chemical Name	Cas. No.	Retention Time
**(1)**	Rutin	153-18-4	2.6 min
**(2)**	Quercitrin	522-12-3	4.2 min
**(3)**	Kaempferol	520-18-3	6.0 min

(a) References

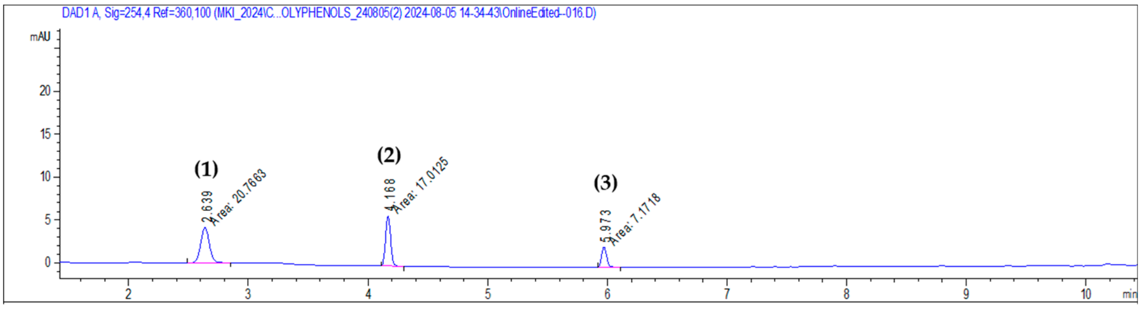

(b) POLE extract

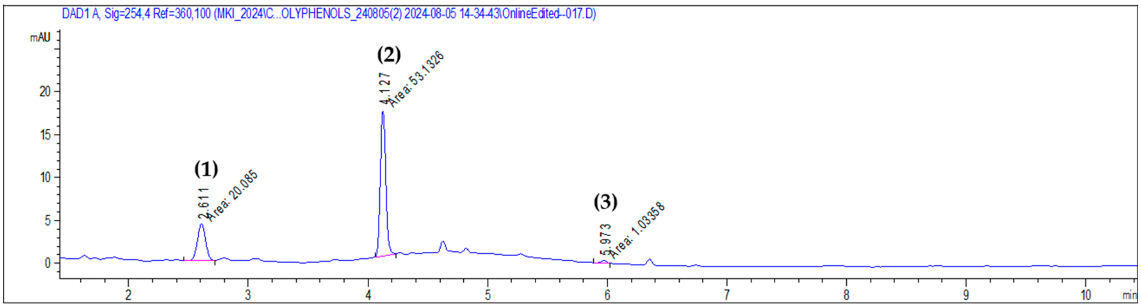

Supplementary Information S2. Taqman probe Assay ID and Amplicon

Gene Name	Assay ID	Interrogated Sequence	Assay Location
GAPDH	4352934E	NM_002046.3	243
KGF (FGF7)	Hs00940253_m1	NM_002009.3	818
IGF-1	Hs01547656_m1	NM_000618.4	489
LEF1	Hs01547250_m1	NM_001130713.2	2110
DKK-1	Hs00183740_m1	NM_012242.2	559
CDKN1A	Hs00355782_m1	NM_000389.4	566
CDKN2A	Hs00923894_m1	NM_000077.4	764
